# Causal relationships between modifiable risk factors and polycystic ovary syndrome: a comprehensive Mendelian randomization study

**DOI:** 10.3389/fendo.2024.1348368

**Published:** 2024-05-08

**Authors:** Yuheng Zhao, Jinglin Pang, Xingyi Fang, Zhaohua Yan, Haili Yang, Qinghua Deng, Tianzhong Ma, Mengqi Lv, Yingying Li, Ziying Tu, Lin Zou

**Affiliations:** ^1^ Department of Reproductive Medicine, Affiliated Hospital of Guangdong Medical University, Zhanjiang, China; ^2^ The First School of Clinical Medicine, Graduate School of Guangdong Medical University, Zhanjiang, China; ^3^ Department of Anorectal Surgery, Affiliated Hospital of Guangdong Medical University, Zhanjiang, China; ^4^ Department of Obstetrics and Gynecology, Affiliated Hospital of Guangdong Medical University, Zhanjiang, China; ^5^ Department of Gynecology, The Second Affiliated Hospital of Guangdong Medical University, Zhanjiang, China; ^6^ Department of Pathology, Southwest Hospital of Army Medical University, Chongqing, China

**Keywords:** polycystic ovary syndrome, modifiable risk factors, Mendelian randomization, lifestyle, inflammation

## Abstract

**Background:**

Polycystic Ovary Syndrome (PCOS) is a heritable condition with an as yet unclear etiology. Various factors, such as genetics, lifestyle, environment, inflammation, insulin resistance, hyperandrogenism, iron metabolism, and gut microbiota, have been proposed as potential contributors to PCOS. Nevertheless, a systematic assessment of modifiable risk factors and their causal effects on PCOS is lacking. This study aims to establish a comprehensive profile of modifiable risk factors for PCOS by utilizing a two-sample Mendelian Randomization (MR) framework.

**Methods:**

After identifying over 400 modifiable risk factors, we employed a two-sample MR approach, including the Inverse Variance Weighted (IVW) method, Weighted Median method, and MR-Egger, to investigate their causal associations with PCOS. The reliability of our estimates underwent rigorous examination through sensitivity analyses, encompassing Cochran’s Q test, MR-Egger intercept analysis, leave-one-out analysis, and funnel plots.

**Results:**

We discovered that factors such as smoking per day, smoking initiation, body mass index, basal metabolic rate, waist-to-hip ratio, whole body fat mass, trunk fat mass, overall health rating, docosahexaenoic acid (DHA) (22:6n-3) in blood, monounsaturated fatty acids, other polyunsaturated fatty acids apart from 18:2 in blood, omega-3 fatty acids, ratio of bisallylic groups to double bonds, omega-9 and saturated fatty acids, total lipids in medium VLDL, phospholipids in medium VLDL, phospholipids in very large HDL, triglycerides in very large HDL, the genus *Oscillibacter*, the genus *Alistipes*, the genus *Ruminiclostridium* 9, the class *Mollicutes*, and the phylum *Tenericutes*, showed a significant effect on heightening genetic susceptibility of PCOS. In contrast, factors including fasting insulin interaction with body mass index, sex hormone-binding globulin, iron, ferritin, SDF1a, college or university degree, years of schooling, household income, the genus *Enterorhabdus*, the family *Bifidobacteriaceae*, the order *Bifidobacteriales*, the class *Actinobacteria*, and the phylum *Actinobacteria* were determined to reduce risk of PCOS.

**Conclusion:**

This study innovatively employs the MR method to assess causal relationships between 400 modifiable risk factors and the susceptibility of PCOS risk. It supports causal links between factors like smoking, BMI, and various blood lipid levels and PCOS. These findings offer novel insights into potential strategies for the management and treatment of PCOS.

## 1 Introduction

Polycystic Ovary Syndrome (PCOS) is a common endocrine metabolic disorder primarily affecting women of reproductive age. The global average prevalence of PCOS is estimated at approximately 6-10% (1). PCOS is characterized by a cluster of interrelated reproductive abnormalities, including disturbances in gonadotropin secretion, increased androgen production, chronic anovulation, and the presence of polycystic ovarian morphology. Commonly associated symptoms encompass hirsutism, acne, alopecia, and weight gain, among others (2). Additionally, PCOS is linked to various clinical complications, such as metabolic disturbances, cardiovascular diseases, diabetes, and psychological disorders. Despite PCOS being a highly heterogeneous condition, the current understanding of its etiology remains incomplete. Genetic and environmental factors, ovarian dysfunction, insulin resistance, iron metabolism, inflammatory responses, hyperandrogenism, hypothalamic-pituitary axis abnormalities and so on may influence the onset of PCOS. Furthermore, gut microbiota may also play a role via the gut-brain axis, potentially triggering PCOS. Hence, elucidating the risk factors for PCOS is imperative and may facilitate early identification and targeted intervention for individuals with PCOS (3–5).

Current observational studies have unveiled numerous modifiable factors associated with PCOS. Lifestyle factors have consistently been recognized as crucial influencers in the etiology of PCOS, among which smoking and alcohol consumption may potentially affect the development of PCOS by influencing steroidogenesis, ovulation, and menstrual cycles (6). Similarly, there are observed connections between dietary imbalances and PCOS (7). In addition, socioeconomic factors, such as educational levels and family income, have been shown to impact PCOS awareness and may increase the risk of PCOS through pathways influencing lifestyle choices (8). Therefore, elaborating on the clear causal effects of lifestyle and social economy on PCOS is helpful for society to coordinate medical resources and macro-control the incidence of PCOS by analyzing the regional economy and lifestyle, ultimately alleviating the social burden. At the same time, the above risk factors often culminate in obesity, a condition highly correlated with PCOS incidence. Although there remains ongoing debate regarding the relationship between central obesity, abdominal obesity, and PCOS (9), obesity can disrupt various metabolic markers, such as insulin resistance and lipid metabolism, leading to chronic inflammation, ovarian tissue damage, and hormonal imbalances (10). Thus, obesity may be critical in the pathogenesis and severity of PCOS, which could be reflected in physical measurements and metabolic features. Additionally, there is a growing focus on micro-level factors, including metabolic indicators, hormones, iron metabolism, inflammatory factors, and gut microbiota. According to existing research, sex hormones are highly related to PCOS, with sex hormone-binding globulin (SHBG) reflecting metabolic alternations as well as total testosterone and androstenedione signifying reproductive dysfunction. Among them, free testosterone is postulated to play a pivotal role in the pathogenic mechanisms underlying all PCOS features (11). Simultaneously, iron metabolism may influence the development of PCOS through pathways such as insulin resistance and inflammatory processes, although there is no consensus regarding the effect of serum iron on PCOS (12). In addition, epidemiological observations imply a potential link between PCOS pathogenesis and the circulating levels of multiple cytokines, with certain circulating inflammatory cells notably elevated in PCOS patients, highlighting the significant role of inflammation in PCOS onset and progression (13). Several research indicates that PCOS is associated with reduced gut microbial diversity compared to healthy individuals, and altered microbial composition suggests a relationship between gut microbiota and PCOS progression. However, the specific genera that exhibit higher abundance in PCOS patients vary across studies (5, 14, 15) Even though various risk factors have been identified in diverse investigations, some continue to be controversial, and certain causalities remain unclear. Further explorations are necessary to determine whether these modifiable factors play causal roles in PCOS development or merely represent comorbid conditions.

However, due to inherent methodological limitations in observational studies, such as confounding and reverse causality, the reported associations fail to elucidate exact causalities. It is infeasible to establish the causal relationship between specific exposures of clear adverse effects on human health with diseases within an experimental setting. To address bias introduced by observational designs, we have employed a novel approach, utilizing Mendelian Randomization (MR) to investigate the causal effects of modifiable factors on PCOS. MR employs genetic variants (Single Nucleotide Polymorphisms, SNPs) as instrumental variables (IVs) to replace exposures (e.g., lifestyle) and outcomes (e.g., PCOS) (16). SNPs are randomly assigned during gamete meiosis and are typically unrelated to other traits. Since genetic variation is fixed and not influenced by postnatal environment, economic factors, social environment, lifestyle habits, and other confounding factors, it cannot be modified. This approach minimizes bias introduced by residual confounding and avoids issues of reverse causality inherent in observational designs. To date, related MR evidence has been limited, hindering a comprehensive understanding of potential causal risk factors for PCOS. In this study, we investigate the causal relationships between eight categories of over 400 suspected major risk factors and PCOS within a two-sample MR framework. Study results are derived from two independent genome-wide association (GWAS) datasets. Clarifying these causal relationships may facilitate early identification and targeted interventions for individuals with PCOS (17).

## 2 Materials and methods

### 2.1 MR design

**Figure 1:**
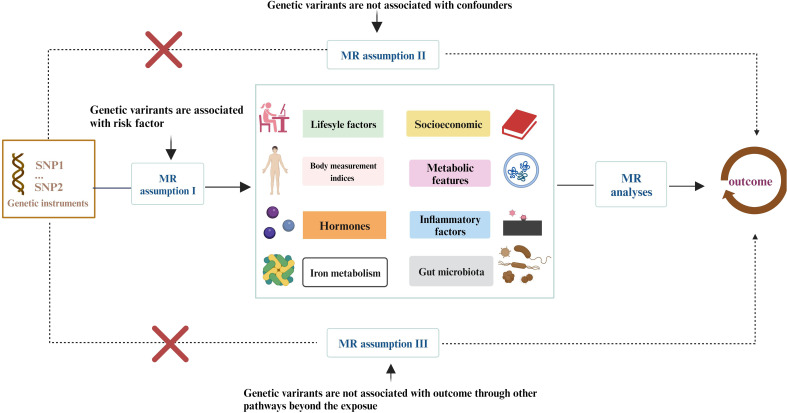
The study encompasses eight domains, including lifestyle behaviors, physical measurements, socioeconomic factors, metabolic indicators, hormones, iron metabolism, inflammatory factors, and gut microbiota. The Mendelian Randomization (MR) framework is based on three fundamental MR assumptions. Please refer to **Supplementary Table 1** for details.

More than 400 critical risk factors were selected and categorized into eight domains (**Figure 1**): lifestyle behaviors, socioeconomic factors, body measurement indices, metabolic indicators, hormones, iron metabolism, inflammatory factors, and gut microbiota. SNPs associated with these risk factors were employed as IVs. The study is based on the three fundamental assumptions of MR research (17): (1) SNPs are closely associated with exposure; (2) SNPs are unrelated to various confounding factors; (3) SNPs influence outcomes solely through exposure. The datasets used in this study were obtained from public databases and had already received prior ethical approval. Therefore, no additional ethical approval was required for this research.

### 2.2 Instrumental variable selection

More than 400 modifiable factors were classified into eight domains and detailed information was displayed in **Supplementary Table 1**. Specifically, the domain of lifestyle factors contained smoking behavior (initiation, daily smoking, and lifetime smoking index), alcohol and coffee consumption, physical activity (moderate to vigorous/intense), insomnia, prolonged sitting, daytime napping, chronic fatigue, snoring, and diet. The domain of socioeconomic factors involved years of education, college degree, unemployment, form of employment in a paid job, household income, and Townsend poverty index. The domain of body measurement indices encompassed body mass index (BMI), basal metabolic rate (BMR), height, waist circumference, body fat percentage, trunk fat mass, whole-body mass and waist-hip ratio(WHR) The domain of metabolic features included overall health rating, type 2 diabetes, fasting blood sugar, glycated hemoglobin A1C (HbA1c), fasting insulin, total cholesterol (TC), triglycerides (TG), high-density lipoprotein (HDL), low-density lipoprotein (LDL) and its subtypes, fatty acids, and amino acids. The domain of hormones comprised dehydroepiandrosterone sulfate (DHEAS), estradiol, cortisol, total testosterone levels, aldosterone, progesterone, 17-Hydroxyprogesterone (17OHP), testosterone, bioavailable testosterone (BT), SHBG levels, leptin, ANDRO, and adiponectin. The domain of iron metabolism contained serum iron, ferritin, total iron binding capacity (TIBC), and transferrin saturation (TSAT). The domain of inflammatory factors included 41 different cytokines and other systemic inflammatory modulators. The domain of gut microbiota encompassed a total of 196 taxonomic groups (classified as 9 Phyla, 16 Classes, 20 Orders, 32 Families, and 119 Genera).

The GWAS dataset was compiled from various consortia sources as follows: (1) Lifestyle: GWAS data for smoking initiation, daily smoking quantity, and intake of alcohol and nicotine were sourced from the Sequencing Consortium of Alcohol and Nicotine Use (18) and the UK Biobank. Genomic analysis of smoking behavior involved information on smoking duration, quantity, and cessation, consolidated into a simulated half-life constant (τ) and a lifetime smoking index (19). Besides, GWAS data for physical activity were obtained from the UK Biobank involving 377,234 individuals, and data for dietary were derived from GWAS performed by Meddens et al (20). (2) Socioeconomic Factors: Data on socioeconomic factors were integrated from European and North American studies including 12 studies conducted at 18 research centers (21). (3) Body Measurement Traits: Genetic variants associated with human body measurement indices (22) were obtained from the GWAS-based GIANT Consortium. For waist circumference (23), genetic variants from the GIANT Consortium involving 224,459 participants were considered. Covariates such as gender, age, age squared, and principal components were used as needed. (4) Metabolites: Large-scale GWAS summary data for 123 circulating metabolic traits from a population of 24,925 individuals in Finland were acquired (24).Data from the Meta-analyses of glucose and insulin-related traits consortium (MAGIC) (25) and the plasma metabolome GWAS dataset (26), studying the relationship between 452 plasma metabolites and lineage variations in 7,824 European participants, were included. (5) Hormone Levels: SNPs associated with TT, BT, and SHBG levels were derived from publicly available summary statistics provided by Ruth et al. using UK Biobank data (27). Summary statistics for DHEAS correlation, including measurements from 9,722 participants (4,308 males and 5,414 females) from the United Kingdom Household Longitudinal Study, were obtained from Prins et al. Cortisol summary statistics were obtained from the CORtisol NETwork (CORNET), involving a meta-analysis of GWAS data from 17 European population cohorts, including 25,314 individuals (28). (6) Iron Homeostasis-Related Metrics: Full-genome association summary data for iron-related traits, including ferritin (N = 246,139), serum iron (N = 163,511), TIBC (N = 135,430), TSAT (N = 131,471), were obtained from the DeCODE Genetics database (29) (7) Systemic Inflammatory Factors: Summary statistics for 41 systemic inflammatory regulatory factors were selected from the latest and largest GWAS (30), encompassing 8,293 Finnish participants from three cohort studies. (8) Gut Microbiota: Genetic IVs for each bacterial taxonomic group were sourced from the largest human gut microbiome element GWAS, involving 18,340 individuals from 24 cohorts, with over 78% of participants having European ancestry (31). Notably, datasets from mixed-race populations were filtered to mitigate population structure bias, and only genetic data from individuals of European Caucasian descent were utilized. The GLGC dataset, the most widely used lipid GWAS dataset, was employed, comprising a sufficiently diverse population with 90.10% of individuals of European descent, thus minimizing potential biases.

### 2.3 GWAS data for PCOS

The FinnGen project, initiated in 2017, represents a collaborative effort involving Finnish universities, research institutions, biopharmaceutical companies, and hospitals. The primary goal of this project is to collect biological samples, genetic information, medical records, and lifestyle data from approximately 500,000 Finnish individuals, constituting roughly one-tenth of the nation’s total population. The GWAS summary data used in our study for PCOS were obtained from the R9 version of the FinnGen project data (https://r9.finngen.fi/). This GWAS dataset encompassed 31,548 cases and 179,322 controls, with adjustments made for age, gender, the top 10 principal components, and genotyping batches during the analysis.The PCOS was diagnozed with ICD-10 code as E28.

### 2.4 Evaluation of the strength of genetic instruments

IVs were selected from related GWAS studies. SNPs reaching genome-wide significance (P < 5 × 10^-8) were extracted. Within a window size of 10,000 kb, SNPs with low linkage disequilibrium (r^2 < 0.01) were chosen to ensure their independence. Furthermore, if selected SNPs were in palindrome sequences or had a minor allele frequency less than 0.01, they would be re-evaluated. To minimize bias from weak IVs, we also calculated the F-statistic representing IV strength. In general, an F-statistic below 10 indicated the presence of weak instrumental variables (**Supplementary Table 2**).

### 2.5 MR analyses and statistical analyses

For each genetic instrument, the calculation of the Wald ratio involved dividing the effect size estimate of the variant’s association with the outcome by the corresponding estimate of the variant’s association with the exposure. When multiple SNPs were available, a meta-analysis of Wald estimates was conducted using the Inverse Variance Weighted (IVW) method. In the absence of horizontal pleiotropy or balanced horizontal pleiotropy, the IVW method provided an unbiased estimate (32). Specifically, Wald ratio estimates were used when the number of SNPs for the trait was fewer than four, while IVW was the primary analysis when the number of SNPs for the IVs exceeded four. Therefore, the main analysis employed the IVW method with random effects (33) to estimate the association between genetic susceptibility and the modifiable risk factors related to PCOS risk. However, it should be noted that the IVW method assumes the absence of heterogeneity or horizontal pleiotropy. Considering that heterogeneity and pleiotropy are significant issues in MR analysis, we used Cochran’s Q statistic to perform a heterogeneity test to evaluate the association between the relevant traits and PCOS risk.Cochran’s Q test was a nonparametric analysis. In our study, we used IVW Cochran’s Q, and a p-value less than 0.05 indicates existence of heterogeneity. Given that the IVW analysis is sensitive to outliers and horizontal pleiotropy, we also conducted two supplementary analyses including the Weighted Median (WM) analysis and the MR-Egger method. The WM model was capable of generating unbiased estimates under the assumption that at least 50% of selected IVs were effective (34). Besides, the MR-Egger regression was used to obtain robust causal estimates in the presence of pleiotropy, with the intercept term testing for horizontal pleiotropy, where an intercept term of zero implied no horizontal pleiotropy. MR-PRESSO was used to identify significant outlier SNPs and produce consistent estimates after removing outlier SNPs, aiming to quantify heterogeneity. The MR-Egger regression intercept and a global test from the MR-PRESSO estimator were used to identify outlier SNPs due to existing heterogeneity. After removing outliers causal effect estimates were recalculated using the IVW method (33). All statistical analyses were performed using R 4.3.1 software packages “TwoSampleMR” and “MRPRESSO.”

## 3 Result

**Figure 2:**
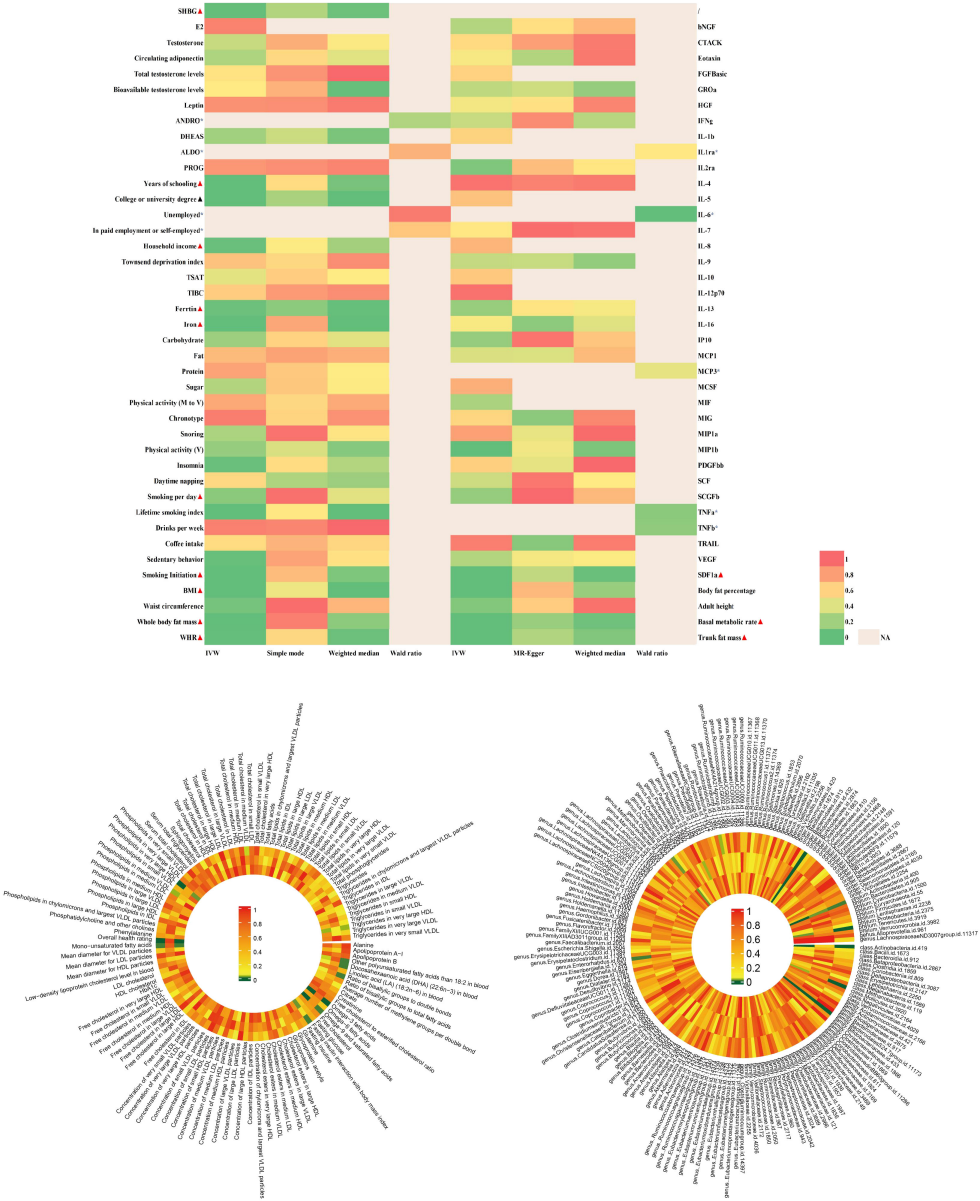
|Analysis of over 400 key risk factors identified in PCOS GWAS. The analysis was conducted within the PCOS-GWAS discovery and is summarized here, including results from five methods, namely IVW, MR-Egger, IVW, MR-Egger, Weighted Median.Detailed statistical information is available in **Supplementary Table 3**. PCOS, Polycystic Ovary Syndrome; GWAS, Genome-Wide Association Study; MR, Mendelian Randomization; IVW, Inverse Variance Weighting; MR-PRESSO, Mendelian Randomization Pleiotropy Residual Sum and Outlier.

**Figure 3:**
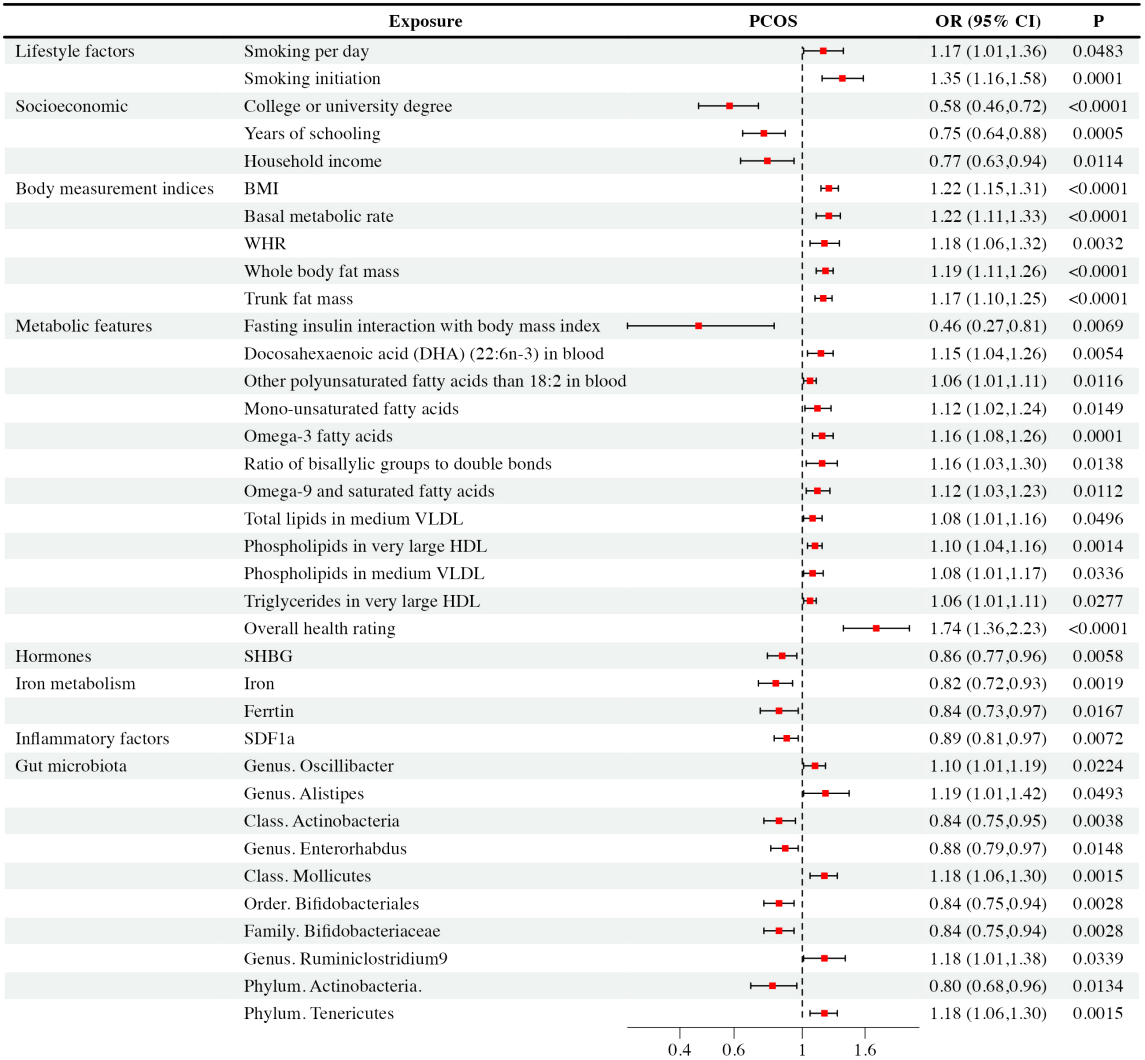
Mendelian randomization analysis positive results forest plot: The analysis is performed in the discovery PCOS GWAS.

The findings of PCOS were summarized in **Figure 2** and elaborated in subsequent sections (**Figure 3** and **Supplementary Table 3**). Positive results were identified for pleiotropy outliers through MR-Egger and MR-PRESSO methods (**Supplementary Table 5**). Scatter plots and funnel plots for SNP exposure associations and SNP-PCOS associations can be found in the appendix.

### 3.1 Association between lifestyle factors and PCOS

Regarding smoking behavior, we observed a causal link between genetic susceptibility of PCOS with the number of cigarettes smoked per day [IVW: OR = 1.17, CI (1.01-1.36), P = 0.48] and smoking initiation [IVW: OR = 1.35, CI (1.16-1.58), P< 0.001]. These findings were consistent across multiple MR methods. Smoking initiation exhibited clear heterogeneity (P = 0.002), but lacked level-specific pleiotropy. Nevertheless, other studied lifestyle factors were not supposed to have causal impacts on PCOS (P > 0.05).

### 3.2 Association between socioeconomic factors and PCOS

Our study revealed that higher education levels, such as obtaining a college or university degree [IVW: OR = 0.58, CI (0.46-0.72), P < 0.001], and longer years of schooling [IVW: OR = 0.75, CI (0.64-0.88), P < 0.001], as well as higher household income [IVW: OR = 0.77, CI (0.63-0.94), P = 0.01], were associated with a reduced risk of PCOS. WM and MR-Egger exhibited consistent results. It’s important to note that college or university degrees, years of schooling, and household income exhibited heterogeneity (P < 0.001, P = 0.043, P = 0.028, respectively), which did not substantially impact the results as random effect IVW could produce robust estimates. We found that they exhibited no level-specific pleiotropy. On the other hand, factors like unemployment, being in paid employment or self-employed, and the Townsend deprivation index showed no significant causal correlation with PCOS and there were no indications of pleiotropy in the observed relationship.

### 3.3 Association between body measurement indices and PCOS

We observed that several body measurement indices, including BMI [IVW: OR = 1.22, CI (1.15-1.31), P < 0.001], BMR [IVW: OR = 1.22, CI (1.11-1.33), P < 0.001], waist-to-hip ratio (WHR) [IVW: OR = 1.18, CI (1.06-1.32), P = 0.003], whole-body mass [IVW: OR = 1.19, CI (1.11-1.26), P < 0.001], and trunk fat mass [IVW: OR = 1.17, CI (1.10-1.25), P < 0.001], exhibited significant associations with an increased risk of PCOS. These findings were consistent across multiple MR methods, including IVW, WM, and MR-Egger. However, it’s worth noting that BMI, BMR, WHR, whole-body fat mass, and trunk fat mass showed evidence of heterogeneity (P < 0.001, P < 0.001, P = 0.002, P < 0.001, and P < 0.001, respectively). Pleiotropy didn’t exist among the results. In contrast, waist circumference, height, and body fat percentage didn’t exhibit significant causal associations with PCOS. Besides, these observed relationship showed no evidence of pleiotropy.

### 3.4 Association between metabolic features and PCOS

Our analysis revealed intriguing associations between metabolic features and PCOS. Specifically, fasting insulin interaction with body mass index showed a negative correlation [IVW: OR = 0.46, CI (0.27-0.81), P = 0.007] with PCOS occurrence. Conversely, overall health rating was associated with an increased risk [IVW: OR = 1.74, CI (1.36-2.23), P < 0.001] of PCOS. Elevated levels of certain fatty acids and lipid components, including DHA [IVW: OR = 1.15, CI (1.04-1.06), P = 0.005], monounsaturated [IVW: OR = 1.12, CI (1.02-1.24), P = 0.012]and other polyunsaturated fatty acids[IVW: OR = 1.06, CI (1.01-1.11), P = 0.005], omega-3 [IVW: OR = 1.16, CI (1.08-1.26), P< 0.001],ratio of bisallylic groups to double bonds [IVW: OR = 1.16, CI (1.03-1.30), P= 0.014] and omega-9 fatty acids [IVW: OR = 1.12, CI (1.03-1.23), P = 0.011], as well as specific lipids in medium VLDL and very large HDL, were positively associated with an increased risk of PCOS. These findings remained consistent across multiple MR methods. Notably, after conducting Cochran’s Q heterogeneity tests, we observed that overall health ratings exhibited heterogeneity (P= 0.003). Furthermore, we did not find level-specific pleiotropy among these factors. In addition, other studied metabolic factors such as tyrosine, type 2 diabetes, valine, HbA1c, histidine, fasting glucose, leucine, TC, sphingomyelins, lactate, phenylalanine, apolipoprotein A-I, apolipoprotein B, 3-hydroxybutyrate, HbA1c, alanine, albumin, acetate, citrate, creatinine, fasting insulin, glucose, glutamine, pyruvate, sphingomyelins, total cholesterol, and triglycerides didn’t exhibit significant causal associations with PCOS. Similarly, the relationship exhibited no signs of pleiotropy.

### 3.5 Association between hormones and PCOS

Among the hormones analyzed, we observed a causal relationship between decreased SHBG levels [IVW: OR = 0.86, CI (0.77-0.96), P = 0.006] and PCOS occurrence, which was further verified via WM and MR-Egger analyses. Although sensitivity analysis revealed pleiotropy (P = 0.04) and heterogeneity(P = 0.02), the result was effectively corrected using MR-PRESSO that excluded rs42374 and ensured the robustness of our results. The analysis indicated that other studied exposures of hormones didn’t show significant associations with PCOS, and there was no pleiotropy found with these relationship.

### 3.6 Association between iron metabolism and PCOS

We found that increased iron levels [IVW: OR = 0.82, CI (0.72-0.93), P = 0.002] and higher ferritin levels [IVW: OR = 0.84, CI (0.73-0.97), P = 0.02] were associated with a reduced risk of PCOS. Sensitivity analysis identified ferritin with pleiotropy (P = 0.018) and heterogeneity (P< 0.001). After correction with MR-PRESSO, which excluded rs1894692 and rs71537957, our results remained robust. However, there was no observed causal correlation between TIBC and TSAT concentrations with PCOS. At the same time, we didn’t detect horizontal pleiotropy in TIBC and TSAT.

### 3.7 Association between inflammatory factors and PCOS

In analyses of 41 inflammatory factors, we found that only SDF1a [IVW: OR = 0.89, CI (0.81-0.97), P = 0.007] exhibited a causal relationship with PCOS. This suggested that an increase in SDF1a concentration was associated with a reduced likelihood of PCOS occurrence. Importantly, there was no pleiotropy or heterogeneity observed with this relationship. The remaining 40 inflammatory factors showed no significant causal association with PCOS. None of them have horizontal pleiotropy.

### 3.6 Association between gut microbiota and PCOS

Based on IVW analysis, we identified significant causal relationships between specific gut microbiota and the risk of PCOS. To be specific, there were five gut microbiota taxa positively associated with PCOS, including the genus *Oscillibacter* [IVW: OR = 1.10, CI (1.01-1.19), P = 0.22], the genus *Alistipes* [IVW: OR = 1.19, CI (1.00-1.42), P = 0.49], the genus *Ruminiclostridium* 9 [IVW: OR = 1.18, CI(1.01-1.38), P = 0.034], the class *Mollicutes* [IVW: OR = 1.18, CI (1.06-1.30), P = 0.001], and the phylum *Tenericutes* [IVW: OR = 1.18, CI (1.06-1.30), P = 0.002]. These findings suggested that higher abundances of the genus *Oscillibacter*, the genus *Alistipes*, the genus *Ruminiclostridium* 9, the class *Mollicutes*, and the phylum *Tenericutes* were causally linked to an increased risk of PCOS. On the contrary, there were another five gut microbiota taxa negatively correlated with PCOS, including the genus *Enterorhabdus* [IVW: OR = 0.88, CI (0.79-0.97), P = 0.015], the family *Bifidobacteriaceae* [IVW: OR = 0.84, CI (0.75-0.94), P = 0.003], the order *Bifidobacteriales* [IVW: OR = 0.84, CI (0.75-0.94), P = 0.003], the class *Actinobacteria* [IVW: OR = 0.84, CI (0.75-0.95), P = 0.004], and the phylum *Actinobacteria* [IVW: OR = 0.80, CI (0.68-0.96), P = 0.133]. Accordingly, higher abundances of the genus *Enterorhabdus*, the family *Bifidobacteriaceae*, the order *Bifidobacteriales*, the class *Actinobacteria*, and the phylum *Actinobacteria* could potentially reduce the risk of PCOS. WM and MR-Egger analyses demonstrated similar results. Although sensitivity analysis revealed significant heterogeneity for the class *Actinobacteria* (P < 0.001), the order *Bifidobacteriales* (P < 0.001), the family *Bifidobacteriaceae* (P < 0.001), and the phylum *Actinobacteria* (P < 0.001), but we did not find level-specific pleiotropy among these factors, random effect IVW could produce robust estimates even under the existence of heterogeneity. Except for the above ten taxa, other gut microbiota genera didn’t show a direct causal relationship with PCOS.

## 4 Discussion

Our MR investigation offered comprehensive insights into potential risk factors for PCOS. We determined that body measurement indices related to obesity, unfavorable lifestyle choices, lipid profiles, unsaturated fatty acids, inflammatory markers, and specific gut microbiota genera were all correlated with an increased susceptibility to PCOS. In contrast, higher levels of education, increased household income, elevated fasting insulin interaction with body mass index, elevated SHBG levels, and specific gut microbiota genera, were associated with a decreased risk of PCOS. However, evidence concerning other factors such as alcohol consumption, coffee consumption, dietary fat, amino acids, physical activity, testosterone, estradiol, TSAT, TIBC, and other parameters in relation to PCOS was inconclusive and necessitated further exploration.

Within lifestyle factors, smoking stands as a significant modifiable risk factor. Legro and colleagues reveal that nearly 40% of PCOS patients are smokers (35). A cohort analysis involving 346 PCOS patients finds a correlation between smoking and increased insulin resistance and elevated free testosterone levels, leading to poorer Homeostatic Model Assessment of Insulin Resistance (HOMA-IR) scores (36). Additionally, a retrospective study discovers an association between increased duration of smoking and altered lipid profiles, elevated white blood cell counts, and decreased prolactin levels in PCOS patients (37).This suggests that smoking may have adverse effects on the biochemical and clinical parameters of PCOS patients. Although the biological mechanisms of smoking in PCOS development are not yet fully elucidated, several studies offer plausible explanations. Nicotine, a primary component of tobacco smoke, can participate in oxidative stress by generating reactive oxygen species and may act as a pro-oxidant in endometrial cells (38). Nicotine exposure can also lead to abnormalities in normal insulin signaling pathways, potentially exacerbating insulin resistance (39). Furthermore, substantial evidence suggests that nicotine and smoking can reduce estrogen levels by inhibiting aromatase activity, thereby potentially affecting metabolic disturbances and hyperandrogenism in PCOS women (40). In contrast to previous MR analyses on smoking and PCOS (41), our study not only established a relationship between smoking initiation and PCOS but also revealed a close association between smoking per day and PCOS. Our research results aligned with most observational studies, providing causal evidence for the link between smoking and PCOS. More importantly, studies have shown that a population’s level of education, economic status, and lifestyle are closely interrelated, but few research focuses on the effect of education level on the risk of PCOS. A cross-sectional study (42) reveals that women with higher levels of education have a significantly better understanding of PCOS compared to those with lower levels of education. Additionally, a case-control study (8) (n=240) finds that low socioeconomic status is associated with an increased risk of PCOS. Our work indicated that longer duration of education, higher educational attainment, and higher family income reduced the risk of PCOS, consistent with existing research. This work elucidated the exact causal role of lifestyle, education level, and economic level on the risk of initiation of PCOS, thus providing direction for the macro-regulation of PCOS in society.

PCOS is also closely associated with obesity characterized by body measurements indices and metabolic features. The close association between obesity and PCOS may be mediated by a variety of mechanisms, such as metabolic changes driven by insulin resistance, steroidogenesis by hyperinsulinemia, and increased adipokine synthesis by subcutaneous and visceral adiposity. A comprehensive meta-analysis of 39 studies (9) has revealed that women with PCOS tend to exhibit abdominal and trunk fat accumulation, while assessments using MRI and CT have shown that the distributions of fat in PCOS and control groups are similar. Hence, the causal relationship between central obesity, trunk obesity, and PCOS remains less definitive. Accumulated evidence from previous observational studies and earlier MR analyses (43, 44) has consistently indicated an increased risk of PCOS with elevated BMI. In our MR study, we not only identified the role of BMI in PCOS risk but also observed consistent positive causal effects of WHR, whole-body fat mass, and trunk fat mass on PCOS, suggesting a continued causal relationship between central obesity and trunk fat and PCOS. In terms of metabolic factors, research has indicated that lipid metabolism abnormalities may not only be contributory to PCOS but also serve as a significant clinical manifestation in patients with PCOS (45). A recent study conducted in Denmark has demonstrated that there are disparities in the median levels of total cholesterol, low-density lipoprotein, and triglycerides in PCOS-afflicted women compared to the control group (46). Meanwhile, a meta-analysis discloses that in addition to known alterations in triglycerides and high-density lipoprotein cholesterol, women with PCOS display elevated levels of low-density lipoprotein cholesterol and non-high-density lipoprotein cholesterol (47). What’s more, approximately 70% of women afflicted with polycystic ovary syndrome have lipid levels at or above critical thresholds. Lipid abnormalities are often concomitant with insulin resistance and heightened cholesterol levels may disrupt hormone synthesis metabolism and exacerbate inflammatory processes, thereby contributing to the inception and progression of PCOS. This work ascertained that elevations in total lipids in medium VLDL, phospholipids in very large HDL, and triglycerides in very large HDL are associated with an increased risk of PCOS. However, total TC, HDL, VLDL, and other key lipid level parameters didn’t manifest significant causal links with PCOS. This discrepancy could be partially attributed to the limited adverse impact of lipid abnormalities, which typically necessitate a prolonged duration to incite metabolic disorders such as cardiovascular diseases and obesity. In addition to lipid metabolism, fatty acid metabolism is intricately linked to the evolution of PCOS. Dietary sources abundant in saturated fatty acids, when compared to monounsaturated and polyunsaturated fatty acids, augment oxidative stress within the body. Additionally, an excess of saturated fatty acids stimulates the production of inflammatory factors in adipose tissue, possessing pro-inflammatory properties that aggravate cellular oxidative stress damage (48), potentially fostering the onset of PCOS, which demonstrates the robustness of our results about the causal effect of fatty acid. Furthermore, insulin resistance (49) is a hallmark clinical feature in PCOS patients, which may culminate in elevated fasting insulin levels, potentially contributing to the development of polycystic follicles and ovulatory complications, thereby exacerbating the clinical presentation of PCOS. This is congruent with our MR analysis, where we observed a conspicuous causal relationship between fasting insulin interaction with body mass index and PCOS.

Regarding associations between hormones and PCOS, SHBG is a sex hormone transport protein produced by the liver. It binds with high affinity to circulating steroids, regulates the concentration of biologically active sex hormones in the bloodstream, and affects their bioavailability. The research suggests that SHBG plays a crucial role in insulin sensitivity, and its decrease may indicate the occurrence of insulin resistance. Metformin can treat insulin resistance in patients with PCOS by increasing SHBG concentrations. Recent studies have indicated that SHBG levels are generally lower in PCOS patients (50, 51). Moreover, research suggests that SHBG inhibits inflammation and lipid accumulation in macrophages and adipocytes, which may be a potential mechanism for the protective role of SHBG in reducing the incidence of metabolic syndrome and its complications (52). Therefore, relatively lower serum SHBG levels have been established as a risk factor for adolescent polycystic ovary syndrome, aligning with our study results, where higher serum levels of SHBG had a protective effect on PCOS, assisting in predicting the occurrence of PCOS. As for iron metabolism, iron is a component of hemoglobin responsible for binding oxygen in red blood cells and transporting oxygen to various tissues in the body. Insufficient serum iron and ferritin may lead to deficient oxygen transport, resulting in metabolic abnormalities and potentially enhanced risk of PCOS. Iron deficiency may disrupt normal levels of sex hormones such as estrogen and testosterone, thereby affecting ovarian function. Additionally, iron deficiency may interfere with energy metabolism and blood sugar regulation, potentially leading to insulin resistance, a common feature of PCOS. Moreover, iron deficiency could increase inflammatory responses, further exacerbating the pathophysiological processes of PCOS. However, a study involving 47 PCOS patients analyzes the serum trace element content of PCOS patients and finds no significant differences in serum iron levels compared with individuals without PCOS (53). Meanwhile, another clinical research including 45 PCOS patients indicates that the serum ferritin levels in the polycystic ovary syndrome group are higher than in the control group, indicating iron overload in PCOS patients (54). Controversy in previous research may be ascribed to limited sample size, distinct populations studied, reverse causality, and potential confounding factors. Since there remains a lack of consensus in clinical discussions regarding the effect of iron metabolism on PCOS, we conducted MR analyses based on public large-scale GWAS data to provide robust results and revealed that elevated levels of serum iron and ferritin had a protective effect on PCOS. Additional research is required to elucidate the potential underlying mechanisms of this connection.

In terms of inflammation factors, epidemiological observational studies suggest that the pathogenesis of PCOS may be related to the levels of various cytokines in circulation, implying that inflammation plays a key role in the onset and progression of PCOS. Observational research indicates that PCOS patients exhibit elevated levels of inflammatory factors in their serum, such as IL-18, TNF-α, IL-4, IL-6, and others (55). Another research indicates that an imbalance in levels of anti-inflammatory and pro-inflammatory cytokines may lead to ovarian dysfunction, alterations in steroid production, and impaired follicle maturation (56). Our MR analysis discovered that SDF1a had a protective effect on PCOS. SDF1a (CXCL12) is a homeostatic chemokine that acts as an anti-inflammatory chemokine during autoimmune inflammatory responses. It contributes to physiological processes such as embryogenesis, hematopoiesis, and angiogenesis (57). Studies suggest that the administration of CXCL12-neutralizing antibodies or small-molecule antagonists of ACKR3 can delay the onset of diseases or prevent disease progression in conditions like cancer, viral infections, inflammatory bowel disease, rheumatoid arthritis, osteoarthritis, and so on (42).Thus, SDF1a may be associated with immune regulation and immune system function, and its elevation may contribute to maintaining normal immune system function, thereby alleviating chronic inflammation, which is in accordance with our results. In addition to inflammation factors, gut microbiota dysbiosis is also a micro-level factors considered as a potential pathogenic factor in PCOS. It has been reported that PCOS patients have altered relative abundances of gut microorganisms, including an increase in *Proteobacteria*, *Escherichia coli/Shigella*, and *Streptococcus*, as well as a decrease in *Akkermansia* and *Lachnospiraceae* (58, 59). Additionally, a cross-sectional study (15) finds that the gut microbiota diversity is lower in the PCOS group compared to the healthy control group, and the Alistipes genus shows significant differences in PCOS. Another study conducted by Zhang (60) finds that in dehydroepiandrosterone (DHEA)-induced PCOS mice, the number of *Chlamydia* increases while the number of *Escherichia coli* decreases, affecting body weight and fat mass. Continuous research suggests that the gut microbiota is closely related to glucose and lipid metabolism, steroid hormone levels, and immune homeostasis, all of which are related to the pathogenesis of PCOS. Our MR analyses indicated the exact causal effect of 196 gut microbiota taxa on the initiation of PCOS, comprehensively revealing the role of gut microbiota in PCOS and providing detailed guidance in monitoring PCOS via gut microbiota. Identifying the relevant risk factors for PCOS not only helps us understand the etiology of PCOS but also allows us to assess the condition of PCOS patients. Additionally, the findings of this study assist clinical healthcare providers in formulating prevention strategies. According to our research results, early prevention of PCOS can be achieved by promoting the adoption of a healthy lifestyle, such as smoking cessation and regular exercise. Besides, increasing medical awareness, especially in rural and lower socioeconomic populations, has become a significant focus for preventing PCOS. Moreover, we recognize that monitoring certain serum markers also has diagnostic and therapeutic significance for PCOS. In terms of treatment, clinical approaches include using metformin can improve insulin resistance, regulate metabolic parameters, and control PCOS. Additionally, the use of oral probiotics, prebiotics, and synbiotics to modulate the gut microbiota is promising in the treatment of PCOS. In conclusion, addressing PCOS risk factors through lifestyle modifications, health awareness campaigns, and targeted therapies can contribute to the prevention and management of this condition. Further research and clinical efforts in these areas are warranted.

This study offers several strengths. Its primary advantage lies in the application of an MR design, allowing for the estimation of causal relationships between two complex heritable traits, and avoiding the inherent biases of traditional observational epidemiological studies. The study incorporates multiple sensitivity analyses to confirm the validity of the instrumental variable assumptions and provides interpretations of results while considering horizontal pleiotropy and outliers. Additionally, this is the first MR analysis that explores a wide range of risk factors, potentially offering new targets for future prevention strategies, which distinguishes it from previous PCOS research databases. A relatively large PCOS database offers a considerable population for research and guarantees the reliability of results. Furthermore, the study rigorously selects IVs and employs multiple sensitivity analyses within the MR analysis, ensuring the fulfillment of the three fundamental MR assumptions. Lastly, the inclusion of predominantly European ancestry participants reduces population structure-related differences. However, some limitations to this study should be noted. Firstly, the study was based on individuals of European descent, population differences may contributed to some discrepancies in results between this work and previous clinical research, thus more diverse analyses in various populations to validate causal relationships and mechanisms are needed. Secondly, several other modifiable risk factors could not be linked to robust genetic IVs, and therefore, they were not included in the study. Global genome-wide studies are supposed to be performed to facilitate MR analyses of these potential risk factors for PCOS. Thirdly, this study is based on summary-level statistics, which limits the exploration of nonlinear relationships between modifiable factors and PCOS and the disease severity. Lastly, the biological mechanisms behind established risk factors of PCOS should be a focus of future research.

## 5 Conclusion

Our study pioneers the application of the MR method to analyze the causal links between 400 modifiable risk factors and PCOS susceptibility. It confirms associations between PCOS and a wide array of factors, such as smoking habits, body composition metrics, blood lipid profiles, gut microbiota composition, and socioeconomic status. These findings provide fresh perspectives on potential approaches for managing and treating PCOS.

## Data availability statement

The original contributions presented in the study are included in the article/**Supplementary Material**. Further inquiries can be directed to the corresponding author.

## Ethics statement

Ethical review and approval can be accessed in the original studies. Informed consent was obtained from all subjects in the original genome-wide association studies. In this MR study, only summary-level statistics were used. No identifiable private information was contained in the GWAS datasets.

## Author contributions

YZ: Conceptualization, Data curation, Formal analysis, Investigation, Methodology, Project administration, Software, Writing – original draft, Validation, Supervision, Visualization. JP: Conceptualization, Data curation, Formal analysis, Writing – original draft, Visualization. XF: Writing – review & editing. ZY: Investigation, Writing – review & editing. HY: Investigation, Writing – review & editing. QD: Investigation, Writing – review & editing. TM: Writing – review & editing. ML: Data curation, Writing – original draft. YL: Formal analysis, Writing – original draft. ZT: Formal analysis, Writing – original draft. LZ: Formal analysis, Writing – review & editing.
